# Qin’s seven steps for endoscopic selective lateral neck dissection via the chest approach in patients with papillary thyroid cancer: experience of 35 cases

**DOI:** 10.1007/s00464-021-08540-9

**Published:** 2021-07-06

**Authors:** Zhen-Xin Chen, Ya-Min Song, Jing-Bao Chen, Xiao-Bo Zhang, Zhan-Hong Lin, Bei-Yuan Cai, Feng-Shun Pang, You Qin

**Affiliations:** Department of Minimally Invasive Surgery, The Second Affiliated Hospital of Guangzhou University of Chinese Medicine (Guangdong Provincial Hospital of TCM), Guangzhou, 510120 People’s Republic of China

**Keywords:** Papillary thyroid cancer, Endoscopic thyroidectomy, Lateral neck dissection, Chest approach, Qin’s seven steps

## Abstract

**Background:**

Endoscopic thyroidectomy is widely performed as it does not result in neck scar. However, there is a paucity of reports pertaining to completely endoscopic lateral neck dissection (LND). In this study, we introduce our step-wise approach for performing endoscopic selective LND via the chest–breast approach. We refer to this approach as Qin’s seven steps.

**Methods:**

The Qin’s seven steps are: (1) establishment of working space range; (2) *dissection of lymph nodes between the SCM and the sternohyoid muscle (level IV)* and exposure of omohyoid; (3) dissection of lymph nodes at level IV; (4) dissection of lymph nodes at level III; (5) *dissection of lymph nodes at carotid triangle (level III);* (6) exposure of accessory nerve and dissection of lymph nodes at level II a; (7) dissection of lymph nodes at level II b. We reviewed the clinical data of 35 patients with papillary thyroid cancer (PTC) who were operated using the Qin’s seven steps.

**Results:**

All 35 patients successfully underwent LND; bilateral LND was performed in 5 patients. The mean tumor size was 1.8 ± 1.0 cm; seven patients had multiple lesions. The mean number of retrieved lymph nodes in level II, III and IV were 8.8 ± 5.6, 6.1 ± 4.0 and 9.3 ± 5.1, respectively. As for complications, there were 3 cases of accessory nerve injury and 1 case of hypoglossal nerve injury. Internal jugular vein injury, cervical plexus injury and lymphatic leakage occurred in 2, 7, and 1 patients, respectively.

**Conclusion:**

The Qin’s seven steps for performing endoscopic selective LND could be safely used in PTC patients with lateral lymph node metastasis. Satisfactory results were achieved in the short-term follow-up period. We recommend the use of Qin’s seven steps for PTC patients who are not desirous of neck scar.

**Supplementary Information:**

The online version contains supplementary material available at 10.1007/s00464-021-08540-9.

The incidence rate of thyroid cancer has steadily increased in recent years; it is currently the fifth most common malignancy in women [1]. Surgery is the preferred treatment for thyroid cancer. However, the traditional open surgery leaves a large and conspicuous scar on the neck. Endoscopic thyroidectomy (ESTC), which does not entail a cervical incision, is therefore preferred by younger patients [2]. The ESTC technique has considerably evolved over the last 20 years [3] and is currently widely used for total thyroidectomy and central lymph nodes dissection [4]. However, over 30% of patients with thyroid cancer have cervical lateral lymph nodes metastasis [5–9]. Lateral neck dissection (LND) is recommended for these patients to improve the prognosis [10, 11]. *However, the traditional open LND necessitates a large neck incision, which has a detrimental effect on the quality of life of patients* [2, 12–14]. A few studies have reported complete endoscopic dissection of the lateral lymph nodes [2, 14, 15]; however, it is a technically challenging procedure that requires surgical expertise. Establishment of standard operating procedures for endoscopic selective LND is a key imperative.

Based on more than 1200 cases of ESTC, we performed endoscopic selective LND in 35 patients with PTC associated with cervical lateral lymph nodes metastasis over the past three years. In order to document the lessons learnt, we summarize the steps of endoscopic selective LND as the Qin’s seven steps. *The Qin’s seven steps are aimed at LND, excluding total thyroidectomy, and central neck dissection.*

## Materials and methods

### Patients and inclusion criteria

Clinical data of 35 patients with PTC who underwent LND at the Guangdong Provincial Hospital of Traditional Chinese Medicine between December 2016 and July 2020 were retrospectively analyzed. The study protocol was approved by the Institutional Review Board (IRB) for ethics at the Guangdong Provincial Hospital of Traditional Chinese Medicine. The inclusion criteria were: (1) confirmed cases of papillary thyroid cancer (PTC) with lateral lymph nodes metastasis (level IIA, IIB, III, or IV) based on ultrasonography, computed tomography (CT), fine needle aspiration (FNA) cytology, or intraoperative exploration; (2) patients who voluntarily opted for endoscopic surgery. The exclusion criteria were: (1) evidence of distant metastasis; (2) level I or V metastatic lymph nodes; (3) invasion of the surrounding tissues; (4) patients with a prior history of surgery or radiotherapy on the neck; and (5) patients who could not tolerate anesthesia or operation.

A total of 35 patients qualified for the study selection criteria were performed endoscopic selective LND at our institution (Table 1). All 35 patients were operated by the same surgeon (You Qin).

**Table 1:** Characteristics of patients with papillary thyroid cancer who underwent lateral neck dissection

No	Sex	Age	Bilateral lateral neck dissection	Operative time (min)	Blood loss (mL)	Tumor size (cm)	Lymph nodes (level)			
							II	III	IV	VI
1	F	39	No	220	200	0.5	1/2	1/2	1/2	0/5
2	F	22	No	375	100	2.2	0/0	2/5	7/9	17/26
3	F	27	No	277	50	2.7	2/11	1/4	0/3	7/12
4	F	48	No	340	30	0.8	0/7	1/8	0/0	0/6
5	F	50	No	285	50	1.2	1/2	0/4	5/10	1/1
6	F	40	No	190	100	0.8	0/1	0/3	0/2	0/4
7	F	33	Yes	410	50	1	3/8 + 2/5	0/4 + 0/2	3/11 + 4/13	4/6 + 2/4
8	F	43	No	320	300	4.3	3/13	0/8	0/13	0/16
9	M	33	No	405	80	2.2	0/19	0/12	1/15	1/16
10	F	55	No	225	50	3	1/6	1/7	1/6	10/14
11	F	35	No	375	100	2	7/10	2/2	2/2	10/13
12	F	43	No	300	20	3	2/11	6/16	0/6	1/5
13	F	37	No	358	20	2.2	0/4	0/5	0/4	0/8
14	F	42	No	265	20	0.7	0/9	0/4	1/10	3/7
15	F	49	No	280	40	3	1/9	0/1	1/13	½
16	F	33	No	245	30	1.6	2/12	0/6	4/17	0/0
17	F	36	No	420	20	1.4	0/12	2/8	5/12	11/12
18	F	50	No	295	50	4.2	4/12	0/1	0/3	¾
19	M	70	No	300	10	3.5	2/7	0/7	8/20	6/6
20	M	64	No	230	30	1.1	3/5	2/9	0/11	1/9
21	M	31	No	330	50	0.9	0/4	0/1	2/10	4/5
22	F	55	No	395	50	1.6	1/6	0/4	5/9	0/1
23	M	23	Yes	395	50	1.4	0/6 + 1/10	1/7 + 2/4	1/4 + 8/20	3/3 + 2/4
24	F	59	No	205	20	0.7	0/9	1/19	0/7	0/8
25	F	47	No	276	30	0.7	2/14	0/7	2/10	5/5
26	M	24	Yes	455	50	1.8	2/18 + 0/29	0/0 + 0/4	0/7 + 2/12	0/6 + 0/8
27	F	30	No	290	30	2.5	3/12	0/3	3/9	8/23
28	M	46	No	195	20	0.9	0/8	1/10	0/8	4/4
29	F	53	Yes	400	50	2.5	1/10 + 1/9	0/2 + 0/3	3/10 + 2/9	1/3 + 1/1
30	F	28	No	275	20	0.5	0/4	3/7	1/4	4/10
31	F	23	Yes	425	100	2.5	2/9 + 1/7	3/7 + 2/6	4/7 + 3/10	4/7 + 5/8
32	F	28	No	280	30	2.6	0/2	0/7	1/17	0/7
33	F	24	No	265	30	3	0/10	2/11	3/10	9/11
34	F	37	No	230	15	0.8	0/5	2/4	4/11	7/9
35	M	53	No	230	30	0.6	0/17	0/7	0/14	0/1

### Operative procedures

#### Thyroidectomy

**Fig. 1:**
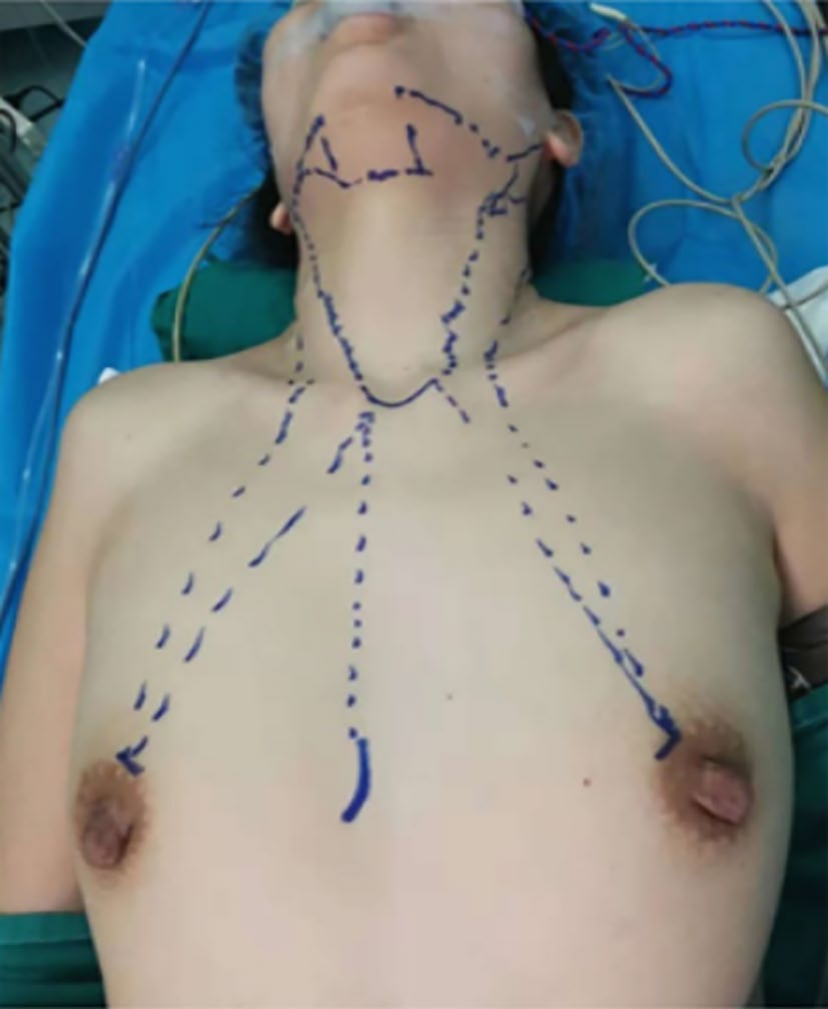
Trocar placement for endoscopic selective LND

A 12-mm incision was made parasternally at the nipple level. Through the incision, approximately 50 ml ‘‘inflation liquid’’ (consisting of 1 mg adrenaline mixed with 500 ml saline) was injected into the subcutaneous deep fascia below the suprasternal notch. A 6-mm incision was made at the 10–11 o’clock positions on the left of the areola and another 6-mm incision was made at the 1–2 o’clock positions on the right of the areola. One 10-mm and two 5-mm trocars were inserted through the 12-mm and 6-mm incisions, respectively (Fig. 1). CO2 gas was insufflated at 6 mmHg pressure with high flow. A 10-mm 30° laparoscope (The laparoscope, television monitor, and equipment for endoscopic thyroid surgery were provided by the Karl-Storz Corporation) was inserted through the 10-mm trocar. The separation of subcutaneous loose connective tissue, establishment of the initial working space, total thyroidectomy and central lymph node dissection were completed in order.

### Endoscopic selective LND—the Qin’s seven steps

**Fig. 2:**
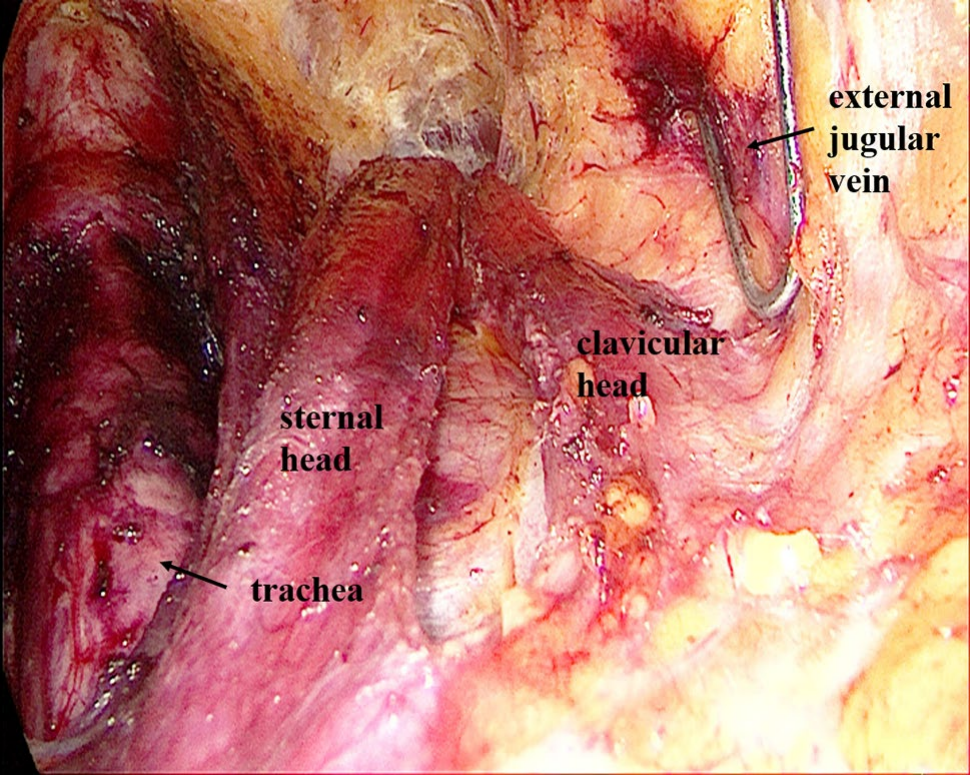
The working space range is dissected to the posterior border of the SCM, to the posterior belly of the digastric muscle

**Fig. 3:**
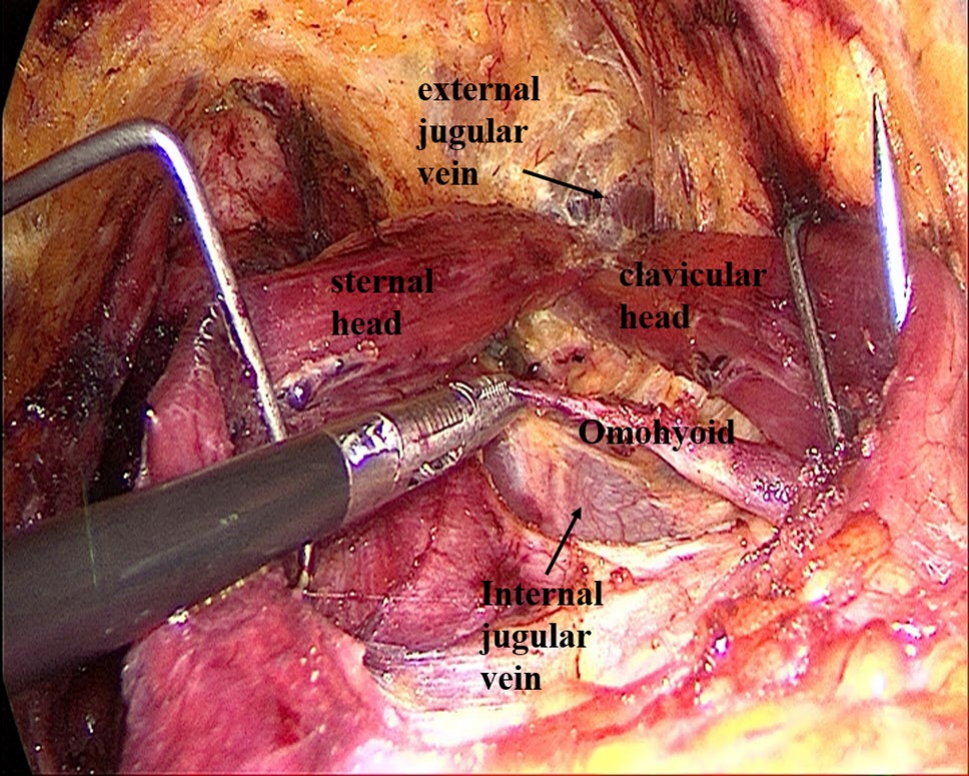
Divide the SCM between the sternal head and the clavicle head, expose the omohyoid and dissect lymph nodes between the SCM and the sternohyoid

**Fig. 4:**
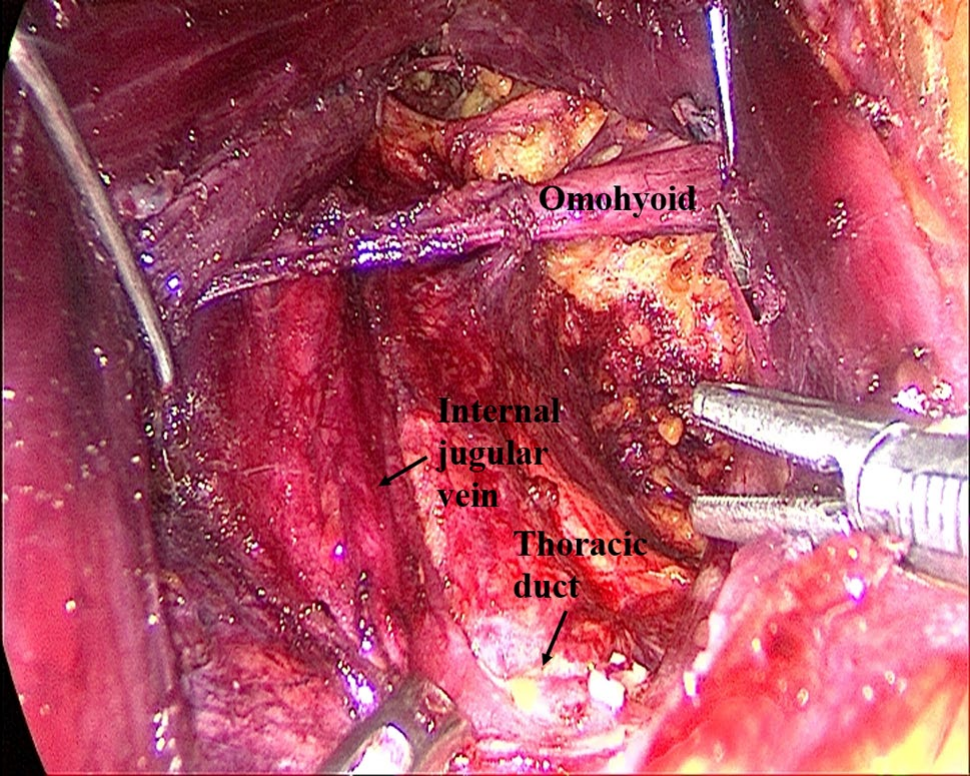
Dissection of lymph nodes at level IV and protection of the transverse cervical artery, phrenic nerve, vagus nerve and carotid artery

**Fig. 5:**
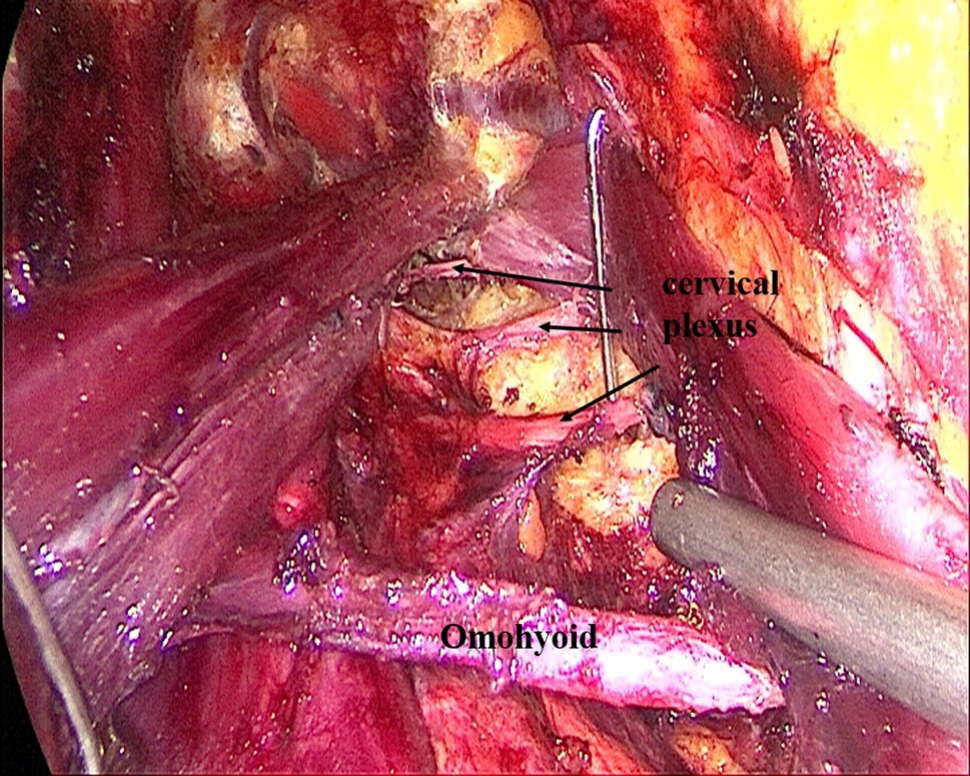
Dissect lymph nodes at level III, especially lymph nodes between the nerve roots and to avoid injury to the cervical plexus

**Fig. 6:**
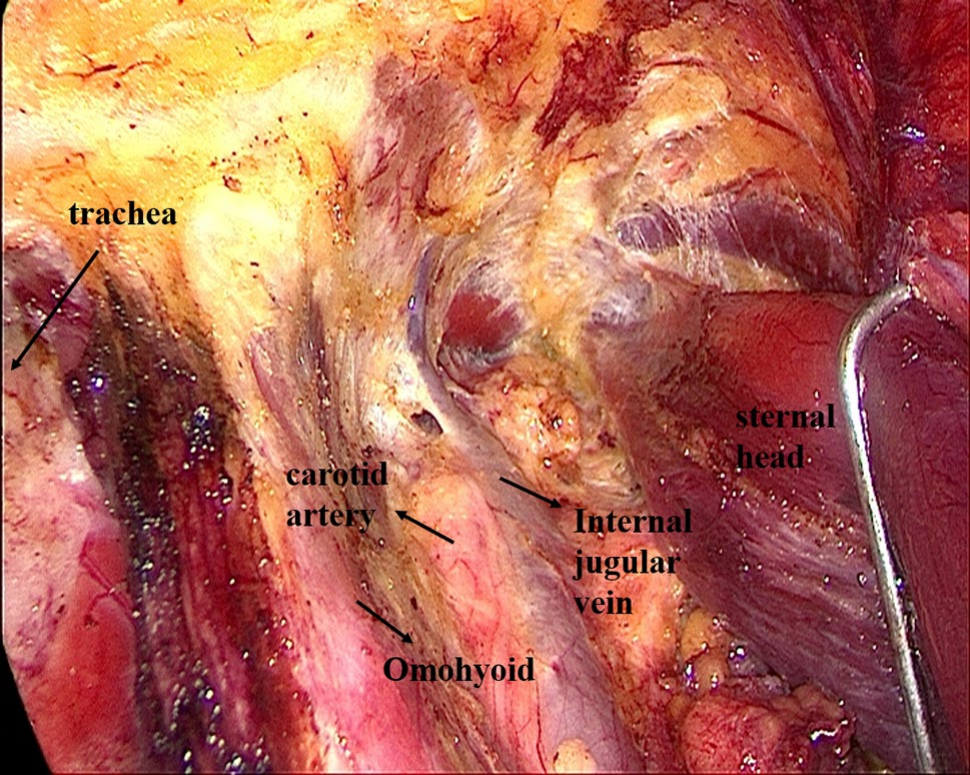
Dissection of lymph nodes at carotid triangle and protection of the hypoglossal nerve

**Fig. 7:**
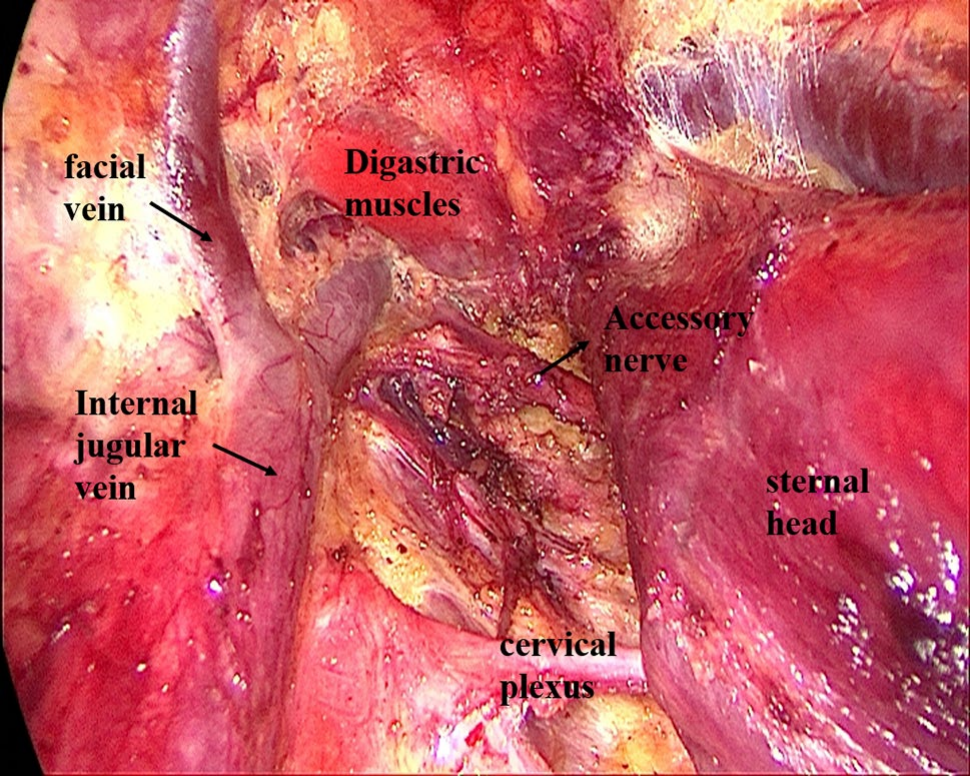
Exposure of accessory nerve and dissection of lymph nodes at level II a and level II b

1. Establishment of working space range
  In the first step, the working space range is dissected to the lateral edge of the posterior border of the sternocleidomastoid (SCM) and to the superior edge of the posterior belly of the digastric muscle. The external jugular vein and cervical plexus are exposed in this step (Fig. 2; Supplemental video. 1). It should be noted that we underline the need for meticulous care to prevent injury to the cervical plexus from the beginning. Subsequently, the anterior border of the SCM is dissected.
2. Dissection of lymph nodes between the SCM and the sternohyoid muscle (level IV) and exposure of omohyoid
  In the second step, the SCM is longitudinally incised at the site between its sternal head and the clavicular head and the incision is extended superiorly to the level of the carotid bifurcation. Two retractors are inserted in the working space from the neck surface to pull the sternal head and the clavicular head of the SCM, respectively. The lymph node between the SCM and the sternohyoid muscle (level IV) is exposed, and the omohyoid and the internal jugular vein are also exposed. The lymph nodes and the surrounding fatty tissue between the SCM and the sternohyoid muscle are freed and cleaned (Fig. 3; Supplemental video. 2). The omohyoid should preferably be preserved in this step, which is different from most previous studies. The internal jugular vein is cautiously dissected up to the cricoid cartilage superiorly and the venous angle inferiorly. During isolation, due care should be taken to ligate the branches of the internal jugular vein, lymph vessels, and the communicating branch of the internal and external jugular veins (if present). Care should be taken to avoid injury to the thoracic duct (left side) or the lymphatic trunk (right side) adjacent to the venous angle.
3. Dissection of lymph nodes at level IV
  In the third step, the internal jugular vein and the sternal head of the SCM are pulled to the tracheal side with a retractor, while the clavicular head of the SCM is pulled to the opposite side. The lymph nodes and the surrounding fatty tissue at the level IV are freed and cleaned. During this step, due care should exercised to protect the *transverse cervical artery* and the phrenic nerve inferiorly, and the vagus nerve and the carotid artery medially (Fig. 4; Supplemental video. 3).
4. Dissection of lymph nodes at level III
  In the fourth step, the retractors are moved up and the sternal head and the clavicular head of the SCM are pulled as above. The lymph nodes and the surrounding fatty tissue at level III are freed and cleaned. During this step, the surgeon should make sure to clean the lymph nodes between the nerve roots and to avoid injury to the cervical plexus (Fig. 5; Supplemental video. 4). In this step, we underline the need for meticulous care to prevent injury to the cervical plexus, which is liable to lead to the loss of cervical skin sensation after operation. Of note, in some cases, there are communicating branches of the internal and external jugular veins, which are vulnerable to injury. This may lead to massive bleeding or even necessitate conversion to open procedure.
5. Dissection of lymph nodes at carotid triangle (level III).
  In the fifth step, the space between the cervical anterior muscles and the SCM muscle is opened. The retractors are moved and used to pull the superior belly of the omohyoid to the trachea side, while the sternal head of the SCM is pulled to the opposite side using another retractor. Lymph nodes at carotid triangle (level III) are exposed and cleaned to the level of the posterior belly of the digastric muscle superiorly. During this step, meticulous attention should be paid to protect the hypoglossal nerve and the facial vein (Fig. 6; Supplemental video. 5). It is unique to define dissection of lymph nodes at carotid triangle as an independent step.
6. Exposure of accessory nerve and dissection of lymph nodes at level II a
  In the sixth step, the clearance of lymph nodes is continued going towards the level II a. Lymph nodes in this compartment should be dissected to the lower edge of the digastric muscle superiorly and to the posterior border of the SCM laterally. The accessory nerve should be isolated and protected during this step. Attention should be paid to prevent injury to the communicating branch between the internal jugular vein and the facial vein besides the accessory nerve; the accessory nerve is particularly liable to be damaged during hemostasis (Fig. 7; Supplemental video. 6). It is a distinctive feature of our approach to emphasize the protection of communicating branches and facial vein. Although the damage of communicating branch and facial vein is not included as a postoperative complication, it has a serious negative impact on endoscopic surgery.
7. Dissection of lymph nodes at level II b
  In the last step, the lymph nodes and the surrounding fatty tissue at the level IIb are freed and cleaned. During this step, it is essential to avoid injury to the parotid gland and the accessory nerve (Fig. 7; Supplemental video. 7). The sequence of dissection of lymph nodes at carotid triangle, level II a, and level II b is different from the previous study [15].

### Post-operative management and follow-up

The drainage tubes are removed when the postoperative drainage volume is less than 30 mL/day and the patients are discharged a day later. Radioactive iodine therapy is administered 3 months after surgery. Patients are followed up at 1, 3, and 6 months after surgery, and annually thereafter.

## Results

Of the 35 patients with PTC who underwent LND (total thyroidectomy and central neck dissection were also performed), 8 patients were male while 27 patients were female. The mean age of patients was 40.3 ± 12.5 years. All patients successfully underwent LND and none of the patients required conversion to the open procedure. Unilateral LND was performed in 30 patients while bilateral LND was performed in 5 patients. The mean tumor size of primary thyroid tumor was 1.8 ± 1.0 cm (range 0.5–4.2). Seven patients had multiple lesions. The mean number of retrieved lymph nodes at level II, III and IV were 8.8 ± 5.6, 6.1 ± 4.0 and 9.3 ± 5.1, respectively. The mean operative time was 307.5 ± 74.4 min (range 190–455). The mean blood loss was 55.0 ± 56.2 mL (range 10–300). As for the complications of LND, 3 patients sustained permanent accessory nerve injury and 1 patient sustained hypoglossal nerve injury. Internal jugular vein injury, cervical plexus injury, and lymphatic leakage occurred in 2, 7, and 1 patient, respectively (Table 2).

**Table 2:** Surgical outcomes of the patients with papillary thyroid cancer who underwent lateral neck dissection

Variable	Value
Age	40.3 ± 12.5
Sex (female/male)	27/8
Tumor size (cm)	1.8 ± 1.0
Hospital stay (days)	5.9 ± 1.5
Drainage time (days)	4.7 ± 1.1
Blood loss (mL)	55.0 ± 56.2
Operation time (mins)	307.5 ± 74.4
No. of retrieved lymph nodes	
II	8.8 ± 5.6
III	6.1 ± 4.0
IV	9.3 ± 5.1
VI	8.0 ± 5.9
Complications of lateral neck dissection	
Lymphatic leakage	1
Cervical plexus injury^a^	7
Accessory nerve injury	3
Hypoglossal nerve injury	1
Internal jugular vein injury	2
Other complications^b^	0

^a^patients with intraoperative cervical plexus transection were defined as cervical plexus injury. ^b^including postoperative bleeding, carotid artery injury, permanent recurrent laryngeal nerve injury and phrenic nerve injury

The mean follow-up period of patients was 18.1 ± 12.0 months. During the follow-up period, no other complications or tumor recurrence were found.

## Discussion

The first ESTC procedure was reported by Huscher et al. in 1997 [3]. Currently, ESTC is a widely accepted procedure. The key advantage of ESTC over open surgery is the absence of visible scar on the neck; this improves the cosmetic results and the quality of life [16–18]. However, there is a paucity of reports concerning completely endoscopic LND due to insufficient exposure and operative difficulties. It is helpful to generalize and standardize the endoscopic LND method by summarizing the operation experience and the procedure. Based on the experience of over 1,200 cases of ESTC, we attempted and explored the endoscopic LND. Till date, we have successfully performed 35 cases of endoscopic LND. In this work, we summarize the surgical procedure, which is referred to as the Qin’s seven steps. Documentation of our approach and the lessons learnt can facilitate mutual learning and communication.

In terms of clinical outcomes, all 35 patients in this study successfully underwent endoscopic LND and none of the patients required conversion to the open procedure. The average number of lymph nodes resected from levels II, III, and IV during endoscopic surgery was similar to that resected during open surgery reported by other researchers [2, 14, 15]. Two patients (5.7%) sustained intraoperative injury to the internal jugular vein, which was successfully repaired endoscopically. Nerve monitoring was used in all 35 patients. Transient recurrent laryngeal nerve palsy occurred in 2 patients (5.7%), while none of the patients developed permanent recurrent laryngeal nerve injury. In addition, there were 3 cases (8.6%) of accessory nerve injury, 1 case (2.9%) of hypoglossal nerve injury, and 1 case (2.9%) of chyle leak. It should be noted that complication rate of accessory nerve injury with 8.6% was relatively high. The complication rate of accessory nerve injury was less than 5% in open LND [2, 19, 20]. The reasons for the high rate of accessory nerve injury in this study can be summarized as follows: Firstly, intraoperative accessory nerve transection (1/35, 2.9%) and postoperative hand lifting difficulty (2/35, 5.7%) were both defined as accessory nerve injury in this study. It is reported that although accessory nerve injury is rarely caused by nerve transection, the acts of dissection and retraction might be sufficient to cause temporary or permanent weakness in up to 20% of patients [11, 21, 22]. Secondly, only 35 cases were included in this study, and small sample size is more likely to lead to data bias. Thirdly, in the first 12 cases of endoscopic LND, lack of experience led to the high complication rate of nerve injuries. In the latter 23 cases, only one case of accessory nerve injury occurred. We believe that with accumulating experience, the rate of nerve injury will gradually decrease and the clinical outcomes of endoscopic surgery for LND are comparable to those of open surgery. This conclusion needs to be further verified in a larger series.

The key limitation of endoscopic surgery via the chest is the difficulty in lymph node dissection at levels I and V; this is a key controversy related to endoscopic LND. Actually, the probability of metastasis and recurrence of thyroid cancer at level I and V is relatively low [11]. According to the diagnosis and treatment standard of thyroid cancer [23], the minimum range of LND includes level IIA, III, and IV. Therefore, we considered that lymph node dissection at level II, III, and IV can meet the treatment requirements in a proportion of patients. Endoscopic surgery is not recommended for thyroid cancer patients with lymph node metastasis at level I or V. We also underline the need for ultrasound and enhanced CT for preoperative evaluation of the metastatic involvement of lymph nodes.

In addition, we have some suggestions for endoscopic LND. Firstly, we believe that endoscopic LND should also be recommended to male patients. According to a previous Chinese study, poor chest wall elasticity and developed neck muscles increase the difficulty of endoscopic LND in male patients. However, we observed no significant differences in clinical outcomes between male and female patients. Secondly, injury of thick vessels can lead to massive bleeding and increase the risk of complications during hemostasis. Therefore, we should pay attention to the protection of facial vein and communicating branches of internal and external jugular veins. Thirdly, we emphasize the dissection of lymph nodes at carotid triangle, which is easy to be ignored. Fourthly, we suggest that level IIA and level IIb lymph node dissection should be carried out in turn after the dissection of lymph nodes at carotid triangle. This sequence is different from that in previous study [15]. We believe that our approach is more conducive to the identification and protection of the accessory nerve. Fifthly, we do not think that it is necessary to incise the omohyoid. Most of previous studies have suggested that the omohyoid should be incised to enlarge the space and facilitate the level III and IV lymph node dissection. We believe we can achieve the same clinical outcomes with the use of the retractors. Sixthly, injury to cervical plexus is liable to lead to the loss of cervical skin sensation after operation. We underline the need for meticulous care to prevent injury to the cervical plexus, especially during the dissection of lymph nodes between cervical plexus at level III. Finally, because of the surgical difficulty and complications, we suggest that endoscopic LND should be performed at centers with rich experience in ESTC. We had performed over 1200 ESTC procedures at our center before performing endoscopic selective LND. However, the mean operative time was 307.5 ± 74.4 min, which is much longer than that of open surgery [2, 15].

In summary, Qin’s seven steps for endoscopic selective LND via the chest approach in patients with differentiated thyroid cancer is a safe approach and achieves good cosmetic results. This technique may offer one more option especially for younger patients.

## Supplementary Information

Below is the link to the electronic supplementary material.

Supplementary file1 (TIF 126 kb)
Fig. R1 Changes in operating time (mins) for each case.

Supplementary file2 (MP4 456002 kb)

Supplementary file3 (MP4 116255 kb)

Supplementary file4 (MP4 166157 kb)

Supplementary file5 (MP4 309761 kb)

Supplementary file6 (MP4 336501 kb)

Supplementary file7 (MP4 383536 kb)

Supplementary file8 (MP4 274762 kb)

Supplementary file9 (MP4 230931 kb)

## References

[1] Bray F, Ferlay J, Soerjomataram I, Siegel RL, Torre LA, Jemal A (2018). Global cancer statistics 2018: GLOBOCAN estimates of incidence and mortality worldwide for 36 cancers in 185 countries. CA Cancer J Clin.

[2] Yan HC, Xiang C, Wang Y, Wang P (2020). Scarless endoscopic thyroidectomy (SET) lateral neck dissection for papillary thyroid carcinoma through breast approach: 10 years of experience. Surg Endosc.

[3] Huscher CS, Chiodini S, Napolitano C, Recher A (1997). Endoscopic right thyroid lobectomy. Surg Endosc.

[4] Kim YS, Joo KH, Park SC, Kim KH, Ahn CH, Kim JS (2014). Endoscopic thyroid surgery via a breast approach: a single institution's experiences. BMC Surg.

[5] Hu D, Zhou J, He W, Peng J, Cao Y, Ren H, Mao Y, Dou Y, Xiong W, Xiao Q, Su X (2018). Risk factors of lateral lymph node metastasis in cN0 papillary thyroid carcinoma. World J Surg Oncol.

[6] Cracchiolo JR, Wong RJ (2018). Management of the lateral neck in well differentiated thyroid cancer. Eur J Surg Oncol.

[7] Ducoudray R, Tresallet C, Godiris-Petit G, Tissier F, Leenhardt L, Menegaux F (2013). Prophylactic lymph node dissection in papillary thyroid carcinoma: is there a place for lateral neck dissection?. World J Surg.

[8] Mulla MG, Knoefel WT, Gilbert J, McGregor A, Schulte KM (2012). Lateral cervical lymph node metastases in papillary thyroid cancer: a systematic review of imaging-guided and prophylactic removal of the lateral compartment. Clin Endocrinol (Oxf).

[9] Ito Y, Tomoda C, Uruno T, Takamura Y, Miya A, Kobayashi K, Matsuzuka F, Kuma K, Miyauchi A (2005). Ultrasonographically and anatomopathologically detectable node metastases in the lateral compartment as indicators of worse relapse-free survival in patients with papillary thyroid carcinoma. World J Surg.

[10] Haugen BR, Alexander EK, Bible KC, Doherty GM, Mandel SJ, Nikiforov YE, Pacini F, Randolph GW, Sawka AM, Schlumberger M, Schuff KG, Sherman SI, Sosa JA, Steward DL, Tuttle RM, Wartofsky L (2016). 2015 American thyroid association management guidelines for adult patients with thyroid nodules and differentiated thyroid cancer: the american thyroid association guidelines task force on thyroid nodules and differentiated thyroid cancer. Thyroid.

[11] Stack BC, Ferris RL, Goldenberg D, Haymart M, Shaha A, Sheth S, Sosa JA, Tufano RP, for the American Thyroid As (2012). American Thyroid Association consensus review and statement regarding the anatomy, terminology, and rationale for lateral neck dissection in differentiated thyroid cancer. Thyroid.

[12] Inoue H, Nibu K, Saito M, Otsuki N, Ishida H, Onitsuka T, Fujii T, Kawabata K, Saikawa M (2006). Quality of life after neck dissection. Arch Otolaryngol Head Neck Surg.

[13] Shah S, Har-El G, Rosenfeld RM (2001). Short-term and long-term quality of life after neck dissection. Head Neck.

[14] Lin P, Liang F, Cai Q, Han P, Chen R, Xiao Z, Wang J, Huang X (2020). Comparative study of gasless endoscopic selective lateral neck dissection via the anterior chest approach versus conventional open surgery for papillary thyroid carcinoma. Surg Endosc.

[15] Guo Y, Qu R, Huo J, Wang C, Hu X, Chen C, Liu D, Chen W, Xiong J (2019). Technique for endoscopic thyroidectomy with selective lateral neck dissection via a chest-breast approach. Surg Endosc.

[16] Sgourakis G, Sotiropoulos GC, Neuhauser M, Musholt TJ, Karaliotas C, Lang H (2008). Comparison between minimally invasive video-assisted thyroidectomy and conventional thyroidectomy: is there any evidence-based information?. Thyroid.

[17] Dobrinja C, Trevisan G, Makovac P, Liguori G (2009). Minimally invasive video-assisted thyroidectomy compared with conventional thyroidectomy in a general surgery department. Surg Endosc.

[18] Gal I, Solymosi T, Szabo Z, Balint A, Bolgar G (2008). Minimally invasive video-assisted thyroidectomy and conventional thyroidectomy: a prospective randomized study. Surg Endosc.

[19] Madenci AL, Caragacianu D, Boeckmann JO, Stack BC, Shin JJ (2014). Lateral neck dissection for well-differentiated thyroid carcinoma: a systematic review. Laryngoscope.

[20] McMullen C, Rocke D, Freeman J (2017). Complications of bilateral neck dissection in thyroid cancer from a single high-volume center. JAMA Otolaryngol Head Neck Surg.

[21] Hillel AD, Kroll H, Dorman J, Medieros J (1989). Radical neck dissection: a subjective and objective evaluation of postoperative disability. J Otolaryngol.

[22] Cappiello J, Piazza C, Nicolai P (2007). The spinal accessory nerve in head and neck surgery. Curr Opin Otolaryngol Head Neck Surg.

[23] CSCO Thyroid Cancer Working Group (2019). Chinese Society of Clinical Oncology (CSCO) diagnosis and treatment guidelines for persistent/recurrent and metastatic differentiated thyroid cancer 2018 (English version). Chin J Cancer Res.

