# Supplementary figures and images for: Qin’s seven steps for endoscopic selective lateral neck dissection via the chest approach in patients with papillary thyroid cancer: experience of 35 cases
Source: Surg Endosc. 2021 Jul 6;36(4):2524–31. doi: 10.1007/s00464-021-08540-9 (PMC8921153; doi:10.1007/s00464-021-08540-9)

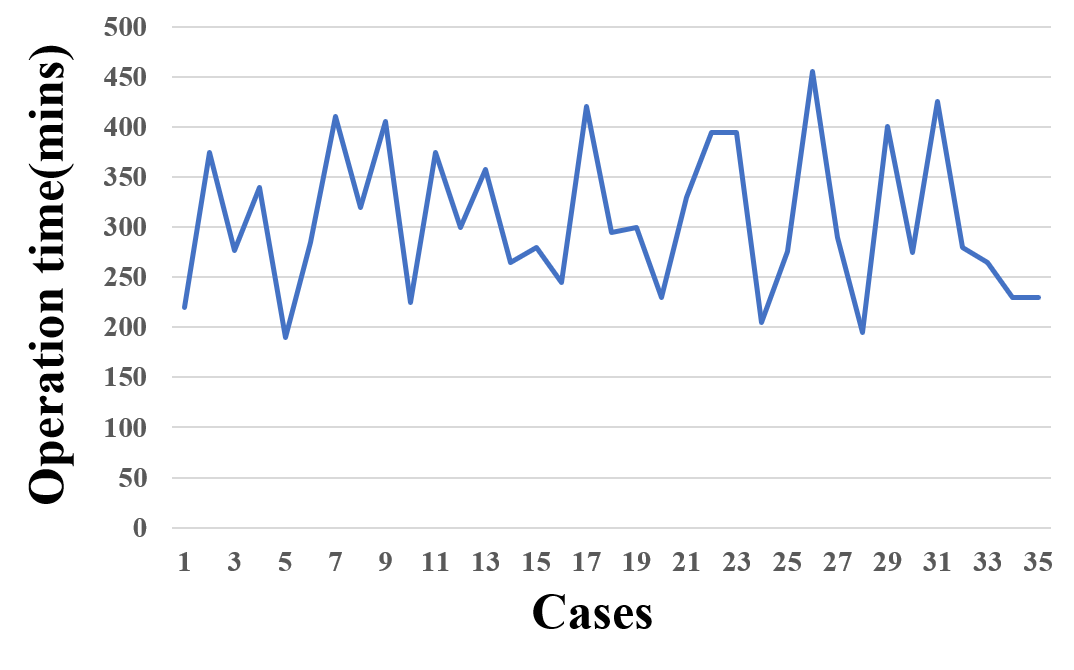

Supplement: Supplementary file 1 — Supplementary file1 (TIF 126 kb) Fig. R1 Changes in operating time (mins) for each case. [file 464_2021_8540_MOESM1_ESM.tif]
